# Efficacy and toxicity of the combination chemotherapy of thalidomide, alkylating agent, and steroid for relapsed/refractory myeloma patients: a report from the Korean Multiple Myeloma Working Party (KMMWP) retrospective study

**DOI:** 10.1002/cam4.970

**Published:** 2016-12-01

**Authors:** Jihyun Kwon, Chang‐Ki Min, Kihyun Kim, Jae‐joon Han, Joon Ho Moon, Hye Jin Kang, Hyeon‐Seok Eom, Min Kyoung Kim, Hyo Jung Kim, Dok Hyun Yoon, Jeong‐Ok Lee, Won Sik Lee, Jae Hoon Lee, Je‐Jung Lee, Yoon‐seok Choi, Sung hyun Kim, Sung‐soo Yoon

**Affiliations:** ^1^ Department of Internal MedicineChungbuk National University HospitalCheongjuKorea; ^2^ Department of Internal MedicineSeoul St. Mary's HospitalThe Catholic University of KoreaKorea; ^3^ Department of Internal MedicineSamsung Medical CenterSungkyunkwan University School of MedicineKorea; ^4^ Departments of Hematology‐OncologySchool of MedicineKyung Hee UniversitySeoulKorea; ^5^ Department of Internal MedicineKyungpook National University HospitalDaeguKorea; ^6^ Department of Internal MedicineKorea Cancer Center HospitalSeoulKorea; ^7^ Hematology‐Oncology ClinicNational Cancer CenterGoyang‐siKorea; ^8^ Department of Internal MedicineYeungnam University Medical CenterDaeguKorea; ^9^ Department of Internal MedicineHallym University Sacred Heart HospitalHallym University College of MedicineAnyangKorea; ^10^ Department of OncologyAsan Medical CenterUniversity of Ulsan, College of MedicineSeoulKorea; ^11^ Department of Internal MedicineSeoul National University Bundang HospitalSeongnamKorea; ^12^ Department of Hemato‐OncologyInje University Busan Paik HospitalBusanKorea; ^13^ Department of Internal MedicineGachon University Gil HospitalIncheonKorea; ^14^ Department of Hematology‐OncologyChonnam National University Hwasun HospitalJeollanamdoKorea; ^15^ Department of Internal MedicineChungnam National University HospitalDaejeonKorea; ^16^ Department of Internal MedicineDong‐A Medical CenterDong‐A University College of MedicineBusanKorea; ^17^ Department of Internal MedicineSeoul National University HospitalSeoulKorea

**Keywords:** Alkylating agent, cyclophosphamide, melphalan, multiple myeloma, thalidomide

## Abstract

We analyzed the treatment responses, toxicities, and survival outcomes of patients with relapsed or refractory multiple myeloma who received daily thalidomide, cyclophosphamide, and dexamethasone (CTD) or daily thalidomide, melphalan, and prednisolone (MTP) at 17 medical centers in Korea. Three‐hundred and seventy‐six patients were enrolled. The combined chemotherapy of thalidomide, corticosteroid, and an alkylating agent (TAS) was second‐line chemotherapy in 142 (37.8%) patients, and third‐line chemotherapy in 135 (35.9%) patients. The response rate overall was 69.4%. Patients who were not treated with bortezomib and lenalidomide before TAS showed a higher response rate compared to those who were exposed to these agents. The estimated median progression‐free survival and overall survival times were 10.4 months and 28.0 months, respectively. The adverse events during TAS were generally tolerable, but 39 (10.4%) patients experienced severe infectious complications. There were no differences in terms of efficacy between CTD and MTP, but infectious complications were more common in CTD group. TAS is an effective treatment regimen which induces a high response rate in relapsed or refractory multiple myeloma patients. Due to the high incidence of grade 3 or 4 infection, proper management of infection is necessary during the TAS treatment, especially the CTD.

## Introduction

Multiple myeloma (MM) remains an incurable hematologic malignancy in spite of the development of various antimyeloma agents. In the early days, alkylating agents such as melphalan or cyclophosphamide were used, but improving the prognosis of MM patients was not satisfactory due to the low efficacy of therapy with an isolated alkylating agent 1. By the 1990s, high‐dose melphalan chemotherapy followed by autologous hematopoietic stem cell transplantation (ASCT) was introduced. This strategy improved the overall response and survival of MM patients, but the benefits of high‐dose chemotherapy and ASCT were limited to younger patients due to the significant toxicity of this therapy 2, 3.

Since the 2000s, there have been major developments in the treatment of MM with the introduction of “novel agents” represented by immunomodulatory drugs (IMiDs) and proteasome inhibitors (PIs). Thalidomide, the first IMiD used to treat myeloma, was demonstrated to have good therapeutic effects in a number of clinical trials. Thalidomide monotherapy or combined therapy with a steroid showed a response rate exceeding 60% in newly diagnosed cases of multiple myeloma 4, 5 and 25–55% in relapsed or refractory cases of myeloma 6, 7, 8, 9, 10, 11, 12, 13. Recently, the combined regimen of thalidomide, an alkylating agent, and a steroid is widely used; it is an active regimen with a higher response rate than thalidomide or alkylating agent monotherapy approaches. The response rate of this combination treatment exceeded 80% in newly diagnosed cases of myeloma 14, 15, 16, 17, 18, and 60–70% in relapsed or refractory cases 19, 20, 21, 22, 23, 24.

The aforementioned results of clinical trials conducted to test thalidomide‐containing multiagent chemotherapy, however, were carried out by targeting small numbers of patients who had been treated only with conventional chemotherapy. At present, more potent drugs such as bortezomib and lenalidomide have been introduced, and increasingly more patients are therefore treated with these highly effective novel agents from the early phase of treatment. It is not known that whether the combination of thalidomide and an alkylating agent is still active in the patients who suffered a recurrence later or failed to show improvement with these newer agents.

The objective of this multicenter retrospective study was to investigate the using pattern, efficacy, and toxicity of a combination of thalidomide, an alkylating agent, and a steroid as chemotherapy in patients with relapsed or refractory multiple myeloma in the era of novel agents. In addition, differences in clinical outcomes according to the choice of the alkylating agent and steroid added to thalidomide were evaluated.

## Method

Seventeen medical centers in the Republic of Korea participated in this multicenter retrospective study. We collected the medical records of patients who were treated with combination chemotherapy including thalidomide, an alkylating agent, and a steroid (TAS) as a form of chemotherapy. All enrolled patients had recurrent and/or refractory multiple myeloma which was defined as relapsed or progressed disease after at least one prior antimyeloma regimen.

TAS included two types of chemotherapy regimens: the cyclophosphamide (intravenously 300 mg/m^2^ for 3 days or orally 50 mg daily), thalidomide (orally 100 mg daily), and dexamethasone (40 mg for 4 days) (CTD) or the melphalan (orally 0.15 mg/kg for 7 days), thalidomide (orally 100 mg daily), prednisolone (60 mg for 7 days) (MTP) regimen. The decision to choose one of the two regimens and to change the dose or schedule of chemotherapy was made by the treating physician in each case.

Clinical data including age, sex, prior therapies, stage according to the International Staging System (ISS), the results of cytogenetics or fluorescence in situ hybridization, baseline laboratory data including white blood cell count, hemoglobin, platelet count, serum calcium, creatinine, and prior treatment lines of antimyeloma therapy were collected. We also analyzed monoclonal protein subtypes, the presence of extramedullary plasmacytoma, myeloma‐related organ dysfunction such as osteolysis, pathologic fractures, and renal replacement by hemodialysis.

Our primary endpoints were the overall response rate to CTD or MTP treatment, and the toxicity profile. Treatment response was evaluated according to the International Myeloma Working Group (IMWG) response criteria 25. The overall response rate was defined as the proportion of patients who showed a partial response or better. Adverse events during TAS were assessed by the National Cancer Institute–Common Terminology Criteria for Adverse Events (NCI CTCAE), version 4.03 26. Secondary endpoints included progression‐free survival (PFS), overall survival (OS), and a comparison of usage patterns and efficacy and toxicity levels between CTD and MTP regimens. PFS was calculated from the time of TAS to the time of disease progression or death, and OS was calculated from the time of TAS to the time of death.

Differences in responses according to patient and disease characteristics were compared by means of a chi‐square analysis. In case of the patients whose best responses to TAS were not evaluable, they were excluded from the analysis of predictive factors for overall response. The median PFS and OS were estimated using the Kaplan–Meier method, and differences between subgroups of patients were compared using log‐rank tests. A multivariate survival analysis was conducted using the Cox proportional hazards model with forward selection with a cutoff of *P < *0.05 to add new variables. SPSS software (version 21; IBM Corp., Armonk, NY) was used for statistical analyses.

This study was supported by the Korean Multiple Myeloma Working Party (KMMWP, Protocol No. KMM158) and reviewed by the Institutional Review Board of each participating medical institutes.

## Results

### Patient characteristics

Baseline characteristics and the appearance of MM‐related organ dysfunctions are described in Table 1. In total, 376 patients were enrolled. Two hundred and thirty‐six patients were treated with CTD and 140 patients with MTP. The median age of the enrolled patients was 66 years old (range 36–90). Data about the initial ISS stage and serum level of B2‐microglobulin were not available in 152 (40.4%) and 138 (36.7%) patients. The most common subtype of the monoclonal heavy chain protein was IgG, and 54 (14.4%) patients had only a light chain. Among the signs of MM‐related organ dysfunction, the most common presentation was osteolytic lesions, followed by anemia. Hypercalcemia and hyperviscosity syndrome were relatively rare. Results of chromosomal analyses or FISH tests were not available in two thirds of all patients. In the remaining patients, *t*(4;14) was reported in 40 (10.6%) patients, *t*(14;16) in 17 (4.5%), del(17p) in 11 (2.9%), *t*(11;14) in 33 (8.8%), and del(13q) in 26 (6.9%) patients.

**Table 1:** Baseline characteristics and multiple myeloma‐related organ failure of patients

	*N* (%)
Sex	
Male	224 (69.6)
Female	152 (40.4)
Age	
Younger than 70	245 (65.2)
70 or older	131 (34.8)
B2‐microglobulin	
<3.5 mg/dL	94 (25.0)
3.5–5.5 mg/dL	58 (15.4)
>5.5 mg/dL	86 (22.9)
Stage by ISS	
I	53 (14.1)
II	81 (21.5)
III	90 (23.9)
Immunophenotype	
Heavy chain	
IgG	183 (48.7)
IgA	100 (26.6)
IgM	9 (2.4)
Light chain	
kappa	208 (55.3)
lambda	154 (41.0)
Anemia (Hb <10 g/dL)	186 (49.8)
Leukopenia (WBC<4 × 10^3^/mm^3^)	88 (23.7)
Thrombocytopenia (platelet <100 × 10^3^/mm^3^)	56 (14.6)
Hypercalcemia (serum Ca>11.5 mg/dL)	14 (3.7)
Renal insufficiency (serum Cr>2.0 mg/dL)	43 (11.4)
Hemodialysis	20 (5.3)
Osteolytic lesions	194 (51.6)
History of fracture	104 (27.7)
Hyperviscosity syndrome	17 (4.5)
Extramedullary plasmacytoma	61 (16.2)

ISS, International Staging System.

The median number of chemotherapy regimens used before receiving TAS was two (range 1–8). TAS was performed as a second‐line therapy in 142 (37.8%) patients and as a third‐line therapy in 135 (35.9%). patients Two hundred and sixty‐six (70.7%) patients were previously treated with bortezomib and 83 (22.1%) patients had been exposed to thalidomide before their chemotherapy, while lenalidomide was used in only 13 (3.5%) patients. Patients who underwent high‐dose chemotherapy followed by ASCT numbered 135, accounting for 35.9% of all patients enrolled in this study.

### Analysis of efficacy and survival rates

The median duration of treatment was 5.3 months (median: six cycles). At the time of the analysis, 13 (3.5%) patients were treated with TAS. The most common cause of cessation was progression of the disease (156 patients, 43.0%), followed by the need for a resting period (62 patients, 17.1%).

The overall response rate was 69.4% (261 patients). CR was confirmed in 61 (16.2%) patients, 5 of whom showed stringent CR. Forty‐three (11.4%) patients showed VGPR, and PR was reported in 157 (41.8%) patients . The best response to TAS was not evaluable in 20 (5.3%) patients. Age, sex, and initial ISS stage did not influence the response to TAS. The subtype of the immunoglobulin heavy chain was not correlated with the response, but patients with a kappa light chain showed higher response rates than those with a lambda light chain (78.3% vs. 66.2%, *P* = 0.009). Patients with anemia (66.7% vs. 80.7%, *P* = 0.002) and thrombocytopenia (57.7% vs. 76.6%, *P* = 0.005) showed significantly lower response rates compared to those without these conditions. Other MM‐related instances of organ failure, including hyperviscosity symptoms, renal insufficiency, osteolytic lesions and/or pathologic fractures, and extramedullary plasmacytoma had no direct correlation with a poor response to TAS.

A history of antimyeloma therapy also affected the response to TAS (Table 2). While exposure to bortezomib and/or thalidomide resulted in a reduced response rate, most of the subgroups showed response rates which exceeded 60%. The overall response rate of patients who were refractory to bortezomib treatment or progressed after prior bortezomib therapy was 69.9%, which was similar with that of bortezomib‐sensitive patients. Response rate of the subgroup of patients who were previously treated with lenalidomide was noticeably low, at less than half (ORR 46.2%), but there were few analyzed patients. Earlier treatment with TAS brought a better response rate. When this therapy was performed as a second‐line therapy, the response rate was significantly higher compared to when it was used as a third‐line therapy or a fourth‐line therapy (*P* = 0.018). A history of high‐dose chemotherapy followed by ASCT had no significant impact on the response rate.

**Table 2:** Response rates according to previous treatment history

	Overall response (CR, VGPR, or PR) *N* (%)	*P*
Previous treatment history		
Bortezomib		
Exposure	177 (70.0)	0.016
No exposure	84 (81.6)	
Thalidomide		
Exposure	52 (65.0)	0.041
No exposure	209 (75.7)	
Lenalidomide		
Exposure	6 (46.2)	0.032
No exposure	255 (74.3)	
High‐dose therapy and ASCT		
Done	90 (69.2)	0.099
Not done	166 (76.1)	
Lines of chemotherapy		
2nd	111 (82.2)	0.018^a^
3rd	87 (68.0)	
4th	39 (65.0)	
5th≤	17 (65.4)	

a Pearson's chi‐square.

**Figure 1:**
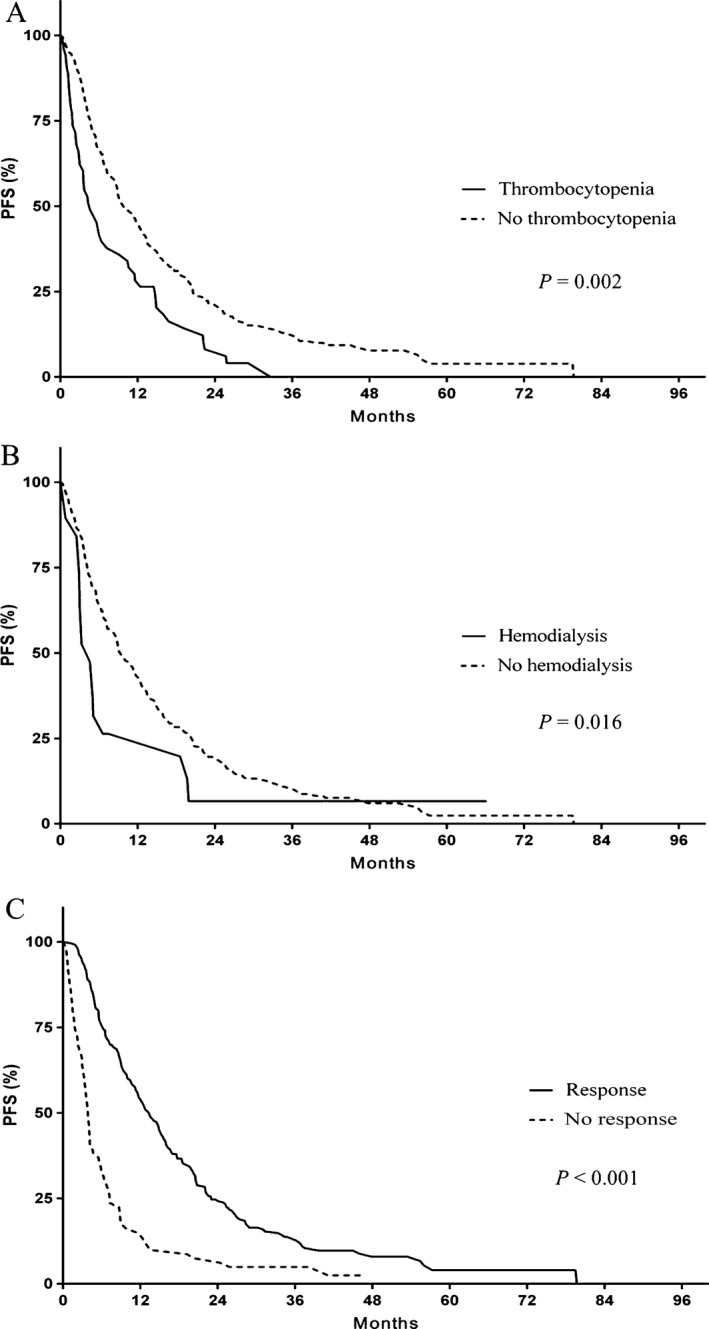
Progression‐free survival (PFS). (A) Presence of thrombocytopenia or not, (B) being on hemodialysis or not, (C) overall response (CR, VGPR, and PR) to study therapy or no response (SD or PD).

**Figure 2:**
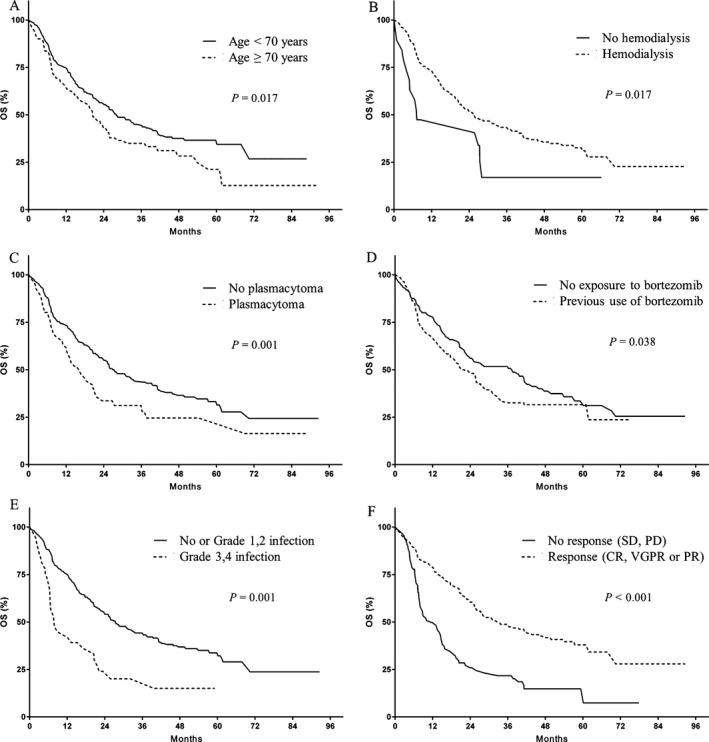
Overall survival (OS). (A) Age younger than 70 years old versus 70 years old or older, (B) being on hemodialysis versus no hemodialysis, (C) presence of extramedullary plasmacytoma versus not, (D) previous exposure to bortezomib versus no exposure, (E) grade 3 or 4 infection during treatment versus no or mild infection, (F) overall response (CR, VGPR, and PR) to study therapy or no response (SD or PD).

Upon a median follow‐up of 22.6 months, 197 (52.4%) patients had died. Median PFS was 10.4 months, and median OS was 28.0 months. In the multivariate analysis, thrombocytopenia (HR: 1.665, *P* = 0.002), hemodialysis (HR: 2.008, *P* = 0.016), and the overall response rates to therapy (HR: 2.871, *P* < 0.001) were found to be significant risk factors for PFS (Fig. 1). Patients who achieved CR, inter alia, showed long PFS of 24.9 months. Prognostic factors for OS included age over 70 years (HR: 1.494, *P* = 0.017), hemodialysis (HR: 2.234, *P* = 0.017), the presence of extramedullary plasmacytoma (HR: 1.852, *P* = 0.001), previous use of bortezomib (HR: 1.467, *P* = 0.038), grade 3 or 4 infection during TAS (HR: 2.005, *P* = 0.001), and an overall response to TAS (HR: 2.806, *P* < 0.001) (Fig. 2).

### Toxicity

Fifty‐six patients stopped TAS due to treatment‐related toxicity (15.6%). Adverse events during TAS are described in Table 3. Most reported cases were mild, at grade 1 or 2. Among nonhematologic adverse events, somnolence and fatigue were the most common symptoms (114 patients, 30.3%). Peripheral neuropathy was reported in 108 (28.7%) patients, but only 11 (2.9%) patients complained of severe symptoms of grade 3 or 4. Hematologic toxicity was reported in 66 (17.6%) patients, and 26 (6.9%) patients had grade 3 or 4 events. The most common hematologic adverse event was neutropenia (44 patients, 11.1%). Among the serious adverse events at grade 3 or 4, the incidence of infection was highest (39 patients, 10.4%), which was the major cause of the cessation of treatment. Three patients died during TAS due to infection; these were one case of pneumonia and two cases of septic shock. All of these three patients were treated with CTD regimen.

**Table 3:** Adverse events

Adverse events	*N* (%)			
	All grade	Grade 3 or 4	Dose reduction of therapy	Discontinuation of therapy
Nonhematologic				
Peripheral neuropathy	108 (28.7)	11 (2.9)	18 (4.8)	17 (4.5)
Venous thrombosis	9 (2.4)	1 (0.3)	0 (0.0)	3 (0.8)
Infection	65 (17.3)	39 (10.4)	2 (0.5)	35 (9.3)
Fatigue & somnolence	114 (30.3)	31 (8.1)	25 (6.6)	24 (6.4)
Dizziness, postural hypotension	44 (11.7)	5 (1.3)	8 (2.1)	5 (1.3)
Gastrointestinal complications	76 (20.2)	2 (0.5)	5 (1.3)	8 (2.1)
Hematologic				
Neutropenia	44 (11.7)	21 (5.6)	9 (2.4)	19 (5.1)
Anemia	31 (8.2)	3 (0.8)	0 (0.0)	2 (0.5)
Thrombocytopenia	17 (4.5)	9 (2.4)	2 (0.5)	3 (0.8)

### Comparison of MTP and CTD

The median age was older in the MTP group (68 years, range 42–90) compared to the CTD group (64 years, 36–84) (*P* < 0.001). Besides, the MTP group contained more elderly patients who were older than 70 (62, 44.3%) compared to the CTD group (69, 29.2%, *P* = 0.003). Patients in the CTD subgroup had more osteolytic lesions (136 patients, 57.6% vs. 58 patients, 41.4%, *P* = 0.011) and instances of extramedullary plasmacytoma (49 patients, 20.7% vs. 12 patients, 8.6%, *P* = 0.004). On the other hand, a history of fracture was more common in the MTP group than the CTD group (47 patients, 33.6% vs. 57 patients, 24.2%, *P* = 0.011). There were no significant differences in the treatment histories between the CTD and MTP groups.

The overall response rates of these two group were similar, 65.0% for the MTP group and 72.0% for the CTD group (*P* = 0.121). Patients who had stopped TAS due to treatment‐related toxicity numbered 40 (16.9%) in the CTD group and 16 (11.4%) in the MTP group. Grade 3 or 4 infection was more common in the CTD group (30 patients, 12.7%) than in the MTP group (nine patients, 6.4%), while grade 3 or 4 peripheral neuropathy was more common in the MTP group (eight patients, 5.7%) than in the CTD group (three patients, 1.3%). There was no significant difference in the PFS and the OS values between these two groups. (Table 4).

**Table 4:** Comparison of response rate and survival outcome according to the treatment regimens

Subtype of TAS	MTP	CTD	*P* value
	*N* = 140	*N* = 236	
Treatment response	*N* of patients (%)	*N* of patients (%)	
Overall response rate	91 (65.0)	170 (72.0)	0.121^a^
Complete response rate	26 (18.6)	35 (14.8)	0.373^a^
Survival	Months (95% CI)	Months (95% CI)	
Median progression‐free survival (PFS)	12.6 (11.0–14.2)	8.7 (6.7–10.6)	0.178^b^
Median overall survival (OS)	33.5 (20.1–46.9)	26.9 (21.7–32.0)	0.588^b^

MTP, melphalan, thalidomide and prednisolone; CTD, cyclophosphamide, thalidomide, and dexamethasone.

a Pearson's chi‐square.

b Kaplan–Meier survival analysis, log‐rank.

## Discussion

Our multicenter, retrospective study showed that a combination of thalidomide, a steroid, and an alkylating agent is an active chemotherapy regimen in cases of relapsed, refractory multiple myeloma. ORR is near 70% of all cases, and the CR rate was 16.2%. Toxicity levels were generally tolerable, and the incidences of severe neurotoxicity and thromboembolism were extremely low.

It was well known that a combination of thalidomide, a steroid, and an alkylating agent is effective in both newly diagnosed and relapsed refractory multiple myeloma patients, but antimyeloma agents more potent than thalidomide, such as bortezomib and lenalidomide, have been introduced recently. Moreover, numerous novel antimyeloma agents including a new class of proteasome inhibitors along with IMiD and anti‐CD138 monoclonal antibodies are currently in development. The role of thalidomide and an alkylating agent has been reduced by degrees, especially as an induction therapy. Similarly in Korea, thalidomide and/or bortezomib‐containing regimen are used as an induction therapy for transplantation‐eligible patients. For transplant‐ineligible patients, bortezomib–melphalan–prednisolone is typical first‐line therapy, and lenalidomide began to use as a second‐line treatment recently. Therefore, a combination of thalidomide and an alkylating agent is mainly used in patients who have relapsed or who have reached refractory status. However, whether this combined therapy is still effective for patients who were heavily treated with various novels agent earlier is not clear.

Our study found a considerable response rate of the combination therapy of thalidomide, a steroid and an alkylating agent, similar or superior to previous results 19, 20, 21, 22, 23, 24. The response rate of patients who were previously treated with bortezomib or thalidomide is significantly lower compared those who had never been exposed to these agents. The diminished efficacy of the combined therapy of thalidomide, a steroid, and an alkylating agent followed by bortezomib appears to be due mainly to a heavy treatment history. It has been suggested that the acquisition of drug resistance and the refractoriness of myeloma cells during prior chemotherapy are the main causes of the failure of salvage chemotherapy 27. Also, our data indicate that cases with a large number of prior treatments are associated with a poor response to TAS. Nevertheless, the response rate of patients with previous treatment history with bortezomib or thalidomide remains considerably high at approximately 60% or more. Besides, bortezomib treatment failure did not compromise the effects of subsequent TAS therapy. As a result, we could regard TAS therapy as a good salvage treatment option for the patients who failed to prior therapy including bortezomib. The patients exposed to lenalidomide showed a relatively low response rate of 46%, but we cannot prove any correlations because of small number of patients. Despite some researchers revealed that no cross‐resistance between different kinds of IMiDs was observed 28, 29, we need more investigation to verify the optimal sequence of several IMiDs including treatment regimens.

Variable factors, including renal failure with hemodialysis, thrombocytopenia, and the degree of the response to TAS have been defined as independent prognostic factors for PFS. Deterioration of renal function is known to be associated with inferior survival and a high incidence of treatment toxicity in previous reports 30, 31. Severe renal failure does not interfere with the effectiveness of drugs in itself, but this condition reflects a high tumor burden of myeloma. Thrombocytopenia, which is associated with decreased bone marrow function, decreases tolerability to antimyeloma agents in patients. Patients with renal failure or thrombocytopenia must receive treatment at a reduced dose and on a disrupted schedule; hence, the benefits of the treatment are greatly limited in these patients. The presence of extramedullary plasmacytoma retains prognostic significance for OS. Extramedullary plasmacytoma is related to a high tumor burden and underlying refractoriness of tumor cells. It has been shown that the response to thalidomide was relatively poor in relation to extramedullary plasmacytoma despite the significant reduction of monoclonal protein 32, 33. Garcia‐Sanz et al. showed that patients with extramedullary myelomatous lesions had shorter survival after CTD treatment 19.

Hematologic toxicity levels were generally mild, while grade 3 or 4 neutropenia was reported in 5.9% of all patients. Infectious complications were reported in 18% of all patients, and the incidence of severe cases (grade 3 or 4) was 10.5%. It is important to note that the majority of these patients with severe infections, including three cases in which the patients died, stopped TAS permanently. We suggest that the presence of infectious complications has important clinical relevance for the combined therapy of thalidomide, a steroid, and an alkylating agent, and that the management of the infection affects the clinical course of the patient. The incidence of peripheral neuropathy was quite high, at 29.5%, while the majority of cases of peripheral neuropathy were mild. Venous thrombosis was rarely observed, at 2.5%, explained partly by the overall risk of overt thromboembolism of Asian patients is lower than in a western population and by the fact that most physicians use a prophylactic anticoagulant or an antiplatelet agent.

We investigated differences in the treatment behavior, efficacy, and toxicities between the CTD and MTP regimens despite the fact that a direct comparison of the two regimens was not possible due to the limitations of this retrospective study. MTP was more frequently used for older patients, likely because it is advantageous given its oral administration of melphalan in comparison with the intravenous administration of cyclophosphamide. There are no differences in response and survival rates between the MTP and CTD regimens; therefore, the choice of the alkylating agent and steroid added to thalidomide appeared to be less important. The interruption of treatment due to toxicity was more common in the CTD group due to the higher incidence of severe infectious complications in CTD than in the MTP group. The immunosuppressive effect of cyclophosphamide is considered to be the main factor behind the high incidence of infection during CTD. In previous reports, researchers suggested that intravenous cyclophosphamide had an immunosuppressive effect 34, and a decrease in the number of CD4‐positive T cells and regulatory T cells was observed in 35 patients treated with the CTD regimen 35. Therefore, prophylaxis and appropriate management of infections are needed for patients who are treated with thalidomide with a steroid and an alkylating agent, especially in conjunction with intravenous cyclophosphamide.

In conclusion, our data showed that the combined treatment of thalidomide with a steroid and an alkylating agent plays a significant role as a salvage therapy for patients with relapsed or refractory myeloma. The previous use of bortezomib, lenalidomide, and high‐dose chemotherapy and ASCT may have contributed to the relatively low response rates found here. This regimen is generally tolerable, but proper care of infectious complications is important with regard to the prognosis of the patient. The efficacy levels of MTP and CTD were identical, but the CTD regimen led to an increase in cases of severe infection.

## Conflict of Interest

None declared.
